# Determinants and prognostic relevance of aortic stiffness in patients with recent ST-elevation myocardial infarction

**DOI:** 10.1007/s10554-021-02383-0

**Published:** 2021-09-02

**Authors:** Ivan Lechner, Martin Reindl, Christina Tiller, Magdalena Holzknecht, Sarah Niederreiter, Agnes Mayr, Gert Klug, Christoph Brenner, Axel Bauer, Bernhard Metzler, Sebastian Johannes Reinstadler

**Affiliations:** 1 University Clinic of Internal Medicine III, Cardiology and Angiology, Medical University of Innsbruck, Anichstrasse 35, 6020 Innsbruck, Austria; 2 Department of Radiology, Medical University of Innsbruck, Anichstrasse 35, 6020 Innsbruck, Austria

**Keywords:** Aortic stiffness, Pulse wave velocity, Cardiac magnetic resonance, Prognosis, ST-segment elevation myocardial infarction, Cardiovascular risk factors

## Abstract

The association between aortic stiffness, cardiovascular risk factors and prognosis in patients with recent ST-elevation myocardial infarction (STEMI) is poorly understood. We analyzed the relationship between cardiovascular risk factors and arterial stiffening and assessed its prognostic significance in patients with recent STEMI. We prospectively enrolled 408 consecutive patients who sustained a first STEMI and underwent primary percutaneous coronary intervention (PPCI). Aortic pulse wave velocity (PWV), the most widely used measure of aortic stiffness, was determined by the transit-time method using velocity-encoded, phase-contrast cardiac magnetic resonance imaging. Patient characteristics were acquired at baseline and major adverse cardiac and cerebrovascular events (MACCE) were assessed at 13 [interquartile range (IQR) 12–31] months. Cox regression- and logistic regression analysis were performed to explore predictors of aortic stiffness and MACCE. Median aortic PWV was 6.6 m/s (IQR 5.6–8.3 m/s). In multivariable analysis, age [odds ratio (OR) 1.10, 95% confidence interval (CI), 1.08–1.14, p < 0.001] and hypertension (OR 2.45, 95% CI, 1.53–3.91, p < 0.001) were independently associated with increased PWV. Sex, diabetes, smoking status, dyslipidemia, and obesity were not significantly associated with PWV in adjusted analysis (all p > 0.05). High PWV significantly and independently predicted occurrence of MACCE in adjusted analysis [hazard ratio (HR) 2.45, 95% CI 1.19–5.04, p = 0.014]. In patients with recent STEMI, the impact of classical cardiovascular risk factors on aortic stiffness is mainly dependent on age and increased blood pressure. Increased aortic stiffness is associated with adverse clinical outcome post-STEMI, suggesting it as a relevant therapeutic target in this population. Trial (NCT04113356).

**Supplementary Information:**

The online version contains supplementary material available at 10.1007/s10554-021-02383-0.

## Background

Aortic pulse wave velocity (PWV) represents the most widely used measure of aortic stiffness. Elevated aortic stiffness, which results in increased central pulse pressure, left ventricular afterload and reduced coronary artery perfusion, was found to independently predict future cardiovascular events and target organ damage in the general population and in patients with different cardiovascular diseases [1, 2]. Consequently, aortic stiffness can be useful in clinical decision making in a variety of clinical scenarios and represents a well-established therapeutic target to reduce the burden of future cardiovascular events. However, data on the exact role and prognostic relevance of increased aortic stiffness in patients with recent ST-elevation myocardial infarction (STEMI) is sparse [3–5]. Aortic stiffness in STEMI patients is likely to be related to multiple mechanisms including previous exposure to cardiovascular risk factors. However, as yet, there has been no systematic investigation that evaluated the association between aortic stiffness and cardiovascular risk factors for this specific population. Previous studies investigating the relationship between aortic stiffness and cardiovascular risk factors in other populations have reported conflicting results [6, 7]. Evidence for a tight relation between aortic stiffness and age as well as systolic blood pressure is relatively consistent. In contrast, data on the independent association of aortic stiffness with other risk factors such as sex, diabetes, tobacco smoking, dyslipidemia and obesity are not univocal [7, 8]. Consequently, results from these studies cannot be generalized to other populations, including patients with recent STEMI.

Phase-contrast cardiac magnetic resonance (CMR) imaging derived PVW assessment is a validated approach to determine aortic stiffness in vivo [9, 10]. It has been shown to be a robust and reproducible non-invasive technique for the assessment of aortic stiffness in patients with stable coronary artery disease and also after STEMI [2, 11, 12].

The aim of the present study was thus to investigate the association of cardiovascular risk factors with aortic stiffness in a well-defined cohort of patients with recent STEMI, treated by contemporary mechanical reperfusion. Moreover, we explored the prognostic relevance of aortic stiffness in this setting.

## Methods

### Study population and characteristics

In this prospective, observational study, we recruited 408 consecutive STEMI patients in the ‘Magnetic Resonance Imaging In Acute ST-Elevation Myocardial Infarction’ (MARINA-STEMI) trial (NCT04113356).

The inclusion criteria were as follows: (1) first STEMI defined by clinical symptoms suggestive of ischemia and significant ST-segment elevation in at least two contiguous leads (> 0.1 mV in extremity leads; > 0.2 mV in precordial leads), (2) revascularization by primary percutaneous coronary intervention (PPCI) within 24 h after symptom onset, (3) an estimated glomerular filtration rate > 30 ml/min/1.73 m^2^ and (4) Killip class < 3 at time of CMR. Exclusion criteria were age < 18 years, previous coronary infarction and any contraindication to CMR (pacemaker, cerebral aneurysm clip, orbital foreign body, claustrophobia and known contrast agent allergy to gadolinium).

Demographic characteristics, detailed medical history and cardiovascular risk factors (hypertension, diabetes mellitus, smoking status, and hypercholesterolemia) were acquired according to a standardized questionnaire at baseline.

Hypercholesterolemia, diabetes and hypertension was defined as follows: (1) patients had known history of hypercholesterolemia, diabetes or hypertension, (2) patients were on cholesterol-lowering, antidiabetic or antihypertensive medication or (3) hypercholesterolemia, diabetes or hypertension was diagnosed during hospitalization. Biomarker assessment was performed on admission and subsequently once daily, until day 4 [4]. For high-sensitivity cardiac Troponin T, three additional measurements during the first 24 h were performed.

Peak biomarker level was defined as highest concentration during the first 96 h after admission. Peak N-terminal prohormone of brain natriuretic peptide (NT-proBNP) values were missing in 7 (1.7%) patients. Admission glucose levels were missing in 4 (1%) patients.

Written informed consent was obtained from all patients prior study inclusion.

The study was approved by the local ethics committee of the Medical University of Innsbruck and was performed in conformity with the ethical guidelines of the Declaration of Helsinki.

### Clinical endpoints

Major adverse cardiac and cerebrovascular events (MACCE), defined as new congestive heart failure, myocardial re-infarction, stroke and all-cause mortality were assessed by telephone using a standardized questionnaire and all declared endpoints were checked afterwards by carefully reviewing the medical records.

Myocardial re-infarction was defined according to the ACCF/AHA guidelines as ischemic symptoms and/or new significant ST-segment changes accompanied by an increase and/or decrease of high-sensitivity cardiac troponin T (hs-cT) levels. Hs-cT changes were defined as one value being above the 99th percentile of the upper reference limit in patients with normal hs-cT values or a 50% increase in patients with elevated baseline hs-cT values [13]. New congestive heart failure was defined as first onset of cardiac decompensation after discharge of the index event, requiring treatment with intravenous diuretics [13–15]. Stroke was defined according to the updated stroke criteria by a consultant neurologist, as an ischemic or hemorrhagic infarction, resulting in neurological dysfunction [16]. Every patient contributed only once to the composite MACCE endpoint. In patients with more than one event, the most severe endpoint was used (all-cause mortality > myocardial re-infarction > stroke > new congestive heart failure) as previously described [17].

### Cardiac magnetic resonance imaging

CMR scans were performed on a 1.5 Tesla scanner (AVANTO; Siemens, Healthineers AG, Erlangen, Germany). The detailed imaging protocol, post-processing [18], as well as method reproducibility [11] was published previously. Briefly, LV ejection fraction was assessed on short-axis (10–12 slices) cine images using breath-hold, retrospective electrocardiogram (ECG) triggered trueFISP bright-blood sequences. ECG-triggered, phase-sensitive inversion recovery sequences were used to obtain late gadolinium enhancement images 15 min after application of a 0.2 mmol/kg bolus of contrast agent (Gadovist®, Bayer, Leverkusen, Germany). Hyperenhancement was defined by a threshold of five standard deviations higher than the signal intensity of remote myocardium in the opposite LV myocardial segment [19] as described previously [20].

**Fig. 1:**
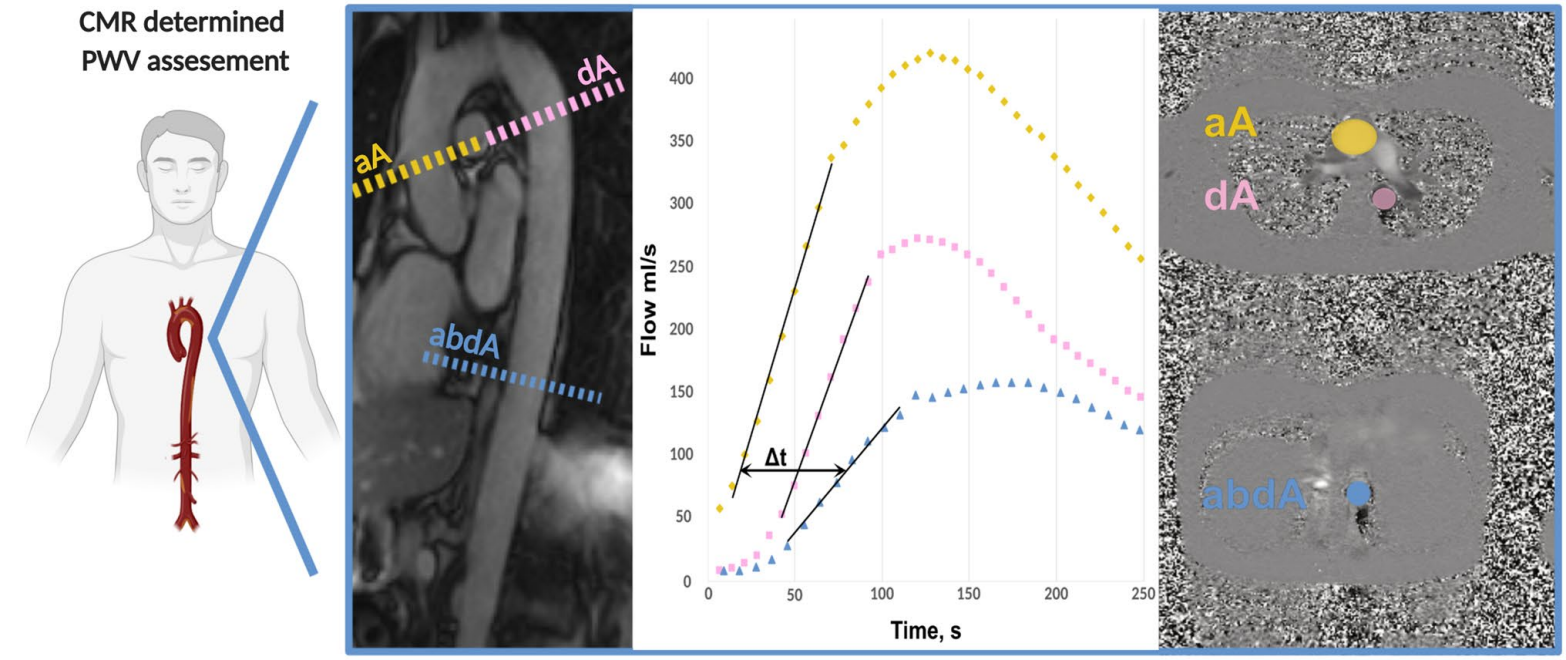
CMR determined PWV assessment. *CMR* cardiac magnetic resonance, *PWV* pulse wave velocity, *aA* ascending aorta, *dA* descending aorta, *abdA* abdominal aorta, *t* time (Created with Biorender)

Pulse wave velocity (PWV) was determined with the use of a velocity-encoded phase-contrast sequence. Velocity encoding was set to 150 cm/s and adjusted in case of aliasing artefacts. Spatial resolution was 1.33 × 1.33 × 8 mm and the repetition time was 13.6 ms. Retrospective ECG triggering with 128 phases per cardiac cycle was used. Acquisition planes were set perpendicular to the ascending and descending thoracic aorta as well as the abdominal aorta. Aortic contours were circled manually (ARGUS; Siemens, Healthineers AG, Erlangen, Germany). Aortic through-plane flow (ml/s) was calculated at all three aortic levels using the velocity values of the velocity-encoded images and displayed in a flow-time diagram. The onset of the systolic upstroke was defined as the ‘arrival’ of the pulse wave at the respective level of measurement [21]. The distance between aortic sites was measured along the aortic luminal midline on an oblique sagittal slice. Finally, PWV was determined by dividing the distance between the ascending and abdominal aorta by the travel time of the pulse wave between sites (Fig. 1).

### Statistical analyses

Statistical analysis was performed by means of IBM SPSS Statistics 26.0. (IBM, Armonk, NY, USA) and MedCalc Version 15.8 (Ostend, Belgium). Continuous variables are presented as mean ± standard deviation or median with interquartile range (IQR) according to their distribution. Categorical variables are shown as frequencies with corresponding percentages. Differences in continuous variables were tested by Wilcoxon–Mann–Whitney-U test. Differences in categorical variables were evaluated by Chi-squared test. To disclose independent predictors of aortic stiffness, possible confounders of aortic stiffness described in previous studies [7] have been entered in a univariable regression analysis. All variables with a p-value < 0.05 in univariable regression analysis were entered in a multivariable linear and binary regression model. PWV was tested as a continuous variable as well as a dichotomized variable. Dichotomization was performed according to median PWV and the optimal cutoff value for MACCE prediction of 7.3 m/s, as observed previously [4]. The relationship between PWV and clinical outcome was expressed by Kaplan–Meier graphs. To reveal independent outcome predictors, possible confounders of aortic stiffness as described in previous studies [7], as well as established prognostic factors in STEMI have been entered in an univariable Cox regression analysis. All variables of interest with a p-value < 0.05 in univariable regression analysis were entered in a multivariable model. For a better comparability, all variables were dichotomized according to median values. All tests were two-tailed and a p-value of < 0.05 was considered as statistically significant.

## Results

### Patient characteristics

A total of 408 patients (16% female) with a median age of 57 (IQR 50–66) years were included in the present study. All patients were treated by PPCI with a median delay of 192 (IQR 125–329) minutes. Time of STEMI to CMR examination was 3 (IQR 2–4) days and there was no significant association between this time and PWV (p = 0.10). Baseline characteristics of the patient population are summarized in Table 1. Median PWV was 6.6 m/s (IQR 5.6–8.3 m/s) in the overall population. Patients with PWV above median were significantly older [64 (IQR 55–71) vs. 52 (IQR 47–57) years, p < 0.001], were more frequently female [n = 44, (21%) vs. n = 23, (12%), p = 0.009], had more often diagnosed hypertension [n = 134, (64%) vs. n = 71, (36%), p < 0.001], and were less likely smoker [n = 93, (45%) vs. n = 130, (65%), p < 0.001]. Patients with higher PWV had higher admission glucose [7.5 (IQR 6.7–9.1) vs. 7.1 mmol/L (IQR 6.3–8.5), p = 0.021], and peak NT-proBNP [1362 (IQR 655–3020) vs. 909 ng/L (IQR 487–1852), p < 0.001] levels, had lower left ventricular ejection fraction [50.8 (IQR 50.8–57.7) vs. 54.2% (IQR 45.7–60.0), p = 0.035], experienced longer delays from symptom onset until PPCI [207 (IQR 136–349) vs. 179 min (IQR 122–309), p = 0.048] and lastly were more likely to suffer from two- [n = 66, (32%) vs. n = 48, (24%)] and three-vessel-disease [n = 32, (15%) vs. n = 14, (7%), p = 0.002].

**Table 1:** Baseline characteristics

	Total population (n = 408)	PWV < 6.6 m/s (n = 200, 49%)	PWV ≥ 6.6 m/s (n = 208, 51%)	*p*-value
Age (years)	57 [50–66]	52 [47–57]	64 [55–71]	** < 0.001**
Female, n (%)	67 (16)	23 (12)	44 (21)	**0.009**
Body mass index, kg/m^2^	26.2 [24.6–28.7]	26.5 [24.7–28.7]	26.2 [24.6–28.7]	0.712
Hypertension, n (%)	203 (50)	71 (36)	134 (64)	** < 0.001**
Antihypertensive medication				
ACE inhibitors, n (%)	47 (12)	13 (7)	34 (16)	**0.002**
ATR blocker, n (%)	41 (10)	14 (7)	27 (13)	**0.046**
Beta blocker, n (%)	41 (10)	11 (6)	30 (14)	**0.003**
Calcium antagonists, n (%)	18 (4)	8 (4)	10 (5)	0.700
Current smoker, n (%)	223 (55)	130 (65)	93 (45)	** < 0.001**
Pack years	20 [0–40]	25 [7–40]	15 [0–40]	**0.021**
Hyperlipidemia, n (%)	222 (54)	107 (54)	115 (55)	0.717
Diabetes mellitus, n (%)	47 (12)	17 (9)	30 (14)	0.061
Admission Glucose (mmol/L)	7.3 [6.4–8.9]	7.1 [6.3–8.5]	7.5 [6.7–9.1]	**0.021**
Culprit lesion, n (%)				0.071
RCA	161 (39)	73 (37)	88 (42)	
LAD	188 (46)	89 (45)	99 (48)	
LCX	55 (14)	36 (18)	19 (9)	
RI	4 (1)	2 (1)	2 (1)	
Number of diseased vessels, n (%)				**0.002**
1	248 (61)	138 (69)	110 (53)	
2	114 (28)	48 (24)	66 (32)	
3	46 (11)	14 (7)	32 (15)	
Prior PCI, n (%)	10 (3)	4 (2)	6 (3)	0.579
Pre-interventional TIMI flow 0, n (%)	255 (63)	118 (59)	137 (66)	0.254
Post-interventional TIMI flow 3, n (%)	352 (86)	180 (90)	172 (83)	0.106
Delay (minutes)	192 [125–329]	179 [122–309]	207 [136–349]	**0.048**
Peak hs-cT (ng/L)	5035 [2115–8920]	4517 [1869–8444]	5366 [2428–9346]	0.066
Peak CK (U/L)	1945 [1006–3532]	1961 [972–3777]	1945 [1091–3337]	0.924
Peak NT-proBNP (ng/L)	1146 [547–2273]	909 [487–1852]	1362 [655–3020]	** < 0.001**
CMR parameters				
PWV (m/s)	6.6 [5.6–8.3]	5.6 [5.2–6.1]	8.3 [7.3–9.9]	** < 0.001**
Time of STEMI to CMR examination (days)	3 [2–4]	3 [2–5]	3 [2–4]	0.10
IS, % LVMM	15.2 [7.0–24.6]	13.9 [6.0–24.4]	16.5 [8.7–25.1]	0.184
LVEF baseline (%)	52.4 [44.8–58.9]	54.2 [45.7–60.0]	50.8 [50.8–57.7]	**0.035**
MVO, n (%)	210 (52)	99 (50)	111 (53)	0.179

All *p*-values < 0.05 are highlighted in bold

*n* number, *RCA* right coronary artery, *LAD* left anterior descending artery, *LCX* left circumflex artery, *RI* ramus intermedius, *PCI* percutaneous coronary intervention, *TIMI* thrombolysis in myocardial infarction, *Hs-cT* high-sensitivity cardiac troponin T, *CK* creatine kinase, *NT-proBNP* N-terminal prohormone of brain natriuretic peptide, *CMR* cardiac magnetic resonance, *PWV* aortic pulse wave velocity, *IS* infarct size, *LVMM* left ventricular mass, *LVEF* left ventricular ejection fraction, *MVO* microvascular obstruction

Antihypertensive medication, including ACE inhibitors [n = 13, (7%) vs. n = 34, (16%), p = 0.002], ATR blocker [n = 14, (7%) vs. n = 27, (13%), p = 0.046] and beta blocker [n = 11, (6%) vs. n = 30, (14%), p = 0.003] were significantly associated to elevated PWV. However, no significant association between calcium antagonists and PWV has been found [n = 8, (4%) vs. n = 10, (5%), p = 0.700].

### Determinants of aortic PWV

In multivariable binary regression analysis, the independent associates of increased PWV (≥ 6.6 m/s) were age [odds ratio (OR) 1.10, 95% confidence interval (CI), 1.08–1.14, p < 0.001], hypertension (OR 2.45, 95% CI, 1.53–3.91, p < 0.001) and number of diseased vessels (OR 1.42, 95% CI, 1.00–2.11, p = 0.049) (Table 2). These findings were similar for age (β = 0.477, p < 0.001), hypertension (β = 0.092, p = 0.036) and number of diseased vessels (β = 0.098, p = 0.024) when PWV was modeled as a continuous variable (multivariable model R = 0.533, p < 0.001) (Table 3). In a further model, where dichotomization at the proposed cut-off value of 7.3 m/s was performed [4], the independent predictors were age (OR 1.12, 95% CI, 1.09–1.15, p < 0.001) and hypertension (OR 2.32. 95% CI, 1.42–3.79, p = 0.001) (Supplementary Table 5). These findings remained significant even after adjustment for time of STEMI to CMR examination.

**Table 2:** Logistic Regression Analysis for Prediction of PWV ≥ 6.6 m/s

	Univariable		Multivariable	
	OR (95% CI)	*p*-value	OR (95% CI)	*p-*value
Age (years)	1.11 (1.09–1.14)	** < 0.001**	1.10 (1.08–1.14)	** < 0.001**
Female sex	0.48 (0.28–0.84)	**0.009**		
Hypertension	3.29 (2.19–4.94)	** < 0.001**	2.45 (1.53–3.91)	** < 0.001**
Current smoker	0.44 (0.29–0.65)	** < 0.001**		
Hyperlipidemia	1.08 (0.73–1.59)	0.717		
Diabetes mellitus	1.81 (0.97–3.41)	0.064		
Peak NT-proBNP	1.00 (1.00–1.00)	**0.003**		
Number of diseased vessels	1.70 (1.27–2.29)	** < 0.001**	1.42 (1.00–2.01)	**0.049**

All *p*-values < 0.05 are highlighted in bold

*PWV* pulse wave velocity, *NT-proBNP* N-terminal prohormone of brain natriuretic peptide, *OR* Odds ratio, *CI* Confidence interval

**Table 3:** Linear Regression Analysis for Prediction of continuous PWV

	Univariable		Multivariable	
	β	*p-*value	β	*p-*value
Age, years	0.513	** < 0.001**	0.477	** < 0.001**
Female sex	− 0.096	0.052		
Hypertension	0.205	** < 0.001**	0.092	**0.036**
Current smoker	− 0.212	** < 0.001**		
Hyperlipidemia	− 0.040	0.415		
Diabetes mellitus	0.052	0.296		
Peak NT-proBNP	0.094	0.061		
Number of diseased vessels	0.177	** < 0.001**	0.098	**0.024**

All *p*-values < 0.05 are highlighted in bold

Multivariable model: R = 0.533, p < 0.001

*PWV* pulse wave velocity, *NT-proBNP* N-terminal prohormone of brain natriuretic peptide

**Fig. 2:**
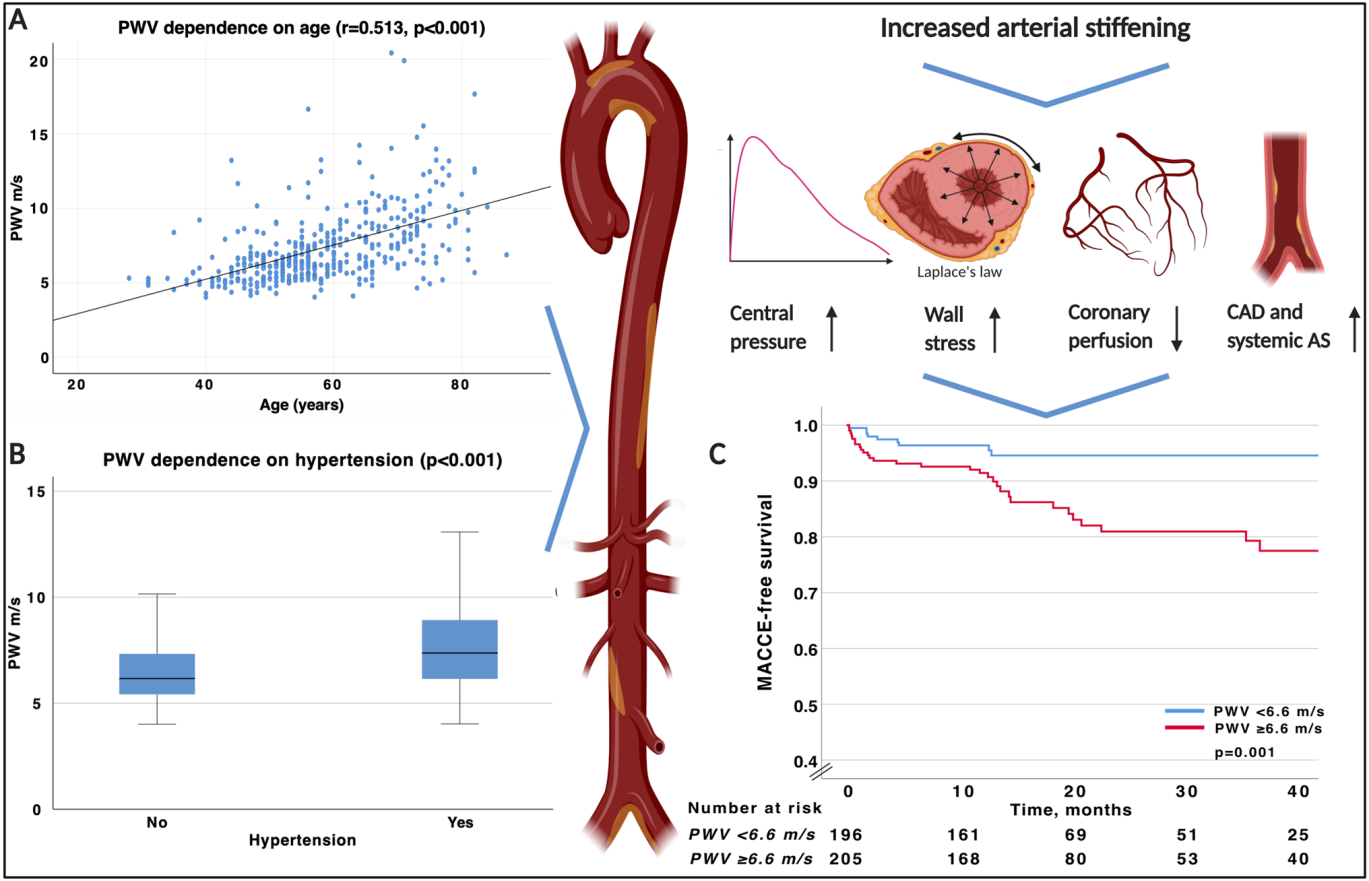
Association of aortic PWV in relation to age, hypertension and clinical outcome. *PWV* pulse wave velocity, *STEMI* ST-elevation myocardial infarction, *CAD* coronary artery disease, *AS* atherosclerosis, *MACCE* major adverse cardiac and cerebrovascular events (Created with Biorender)

The association between PWV and age as well as PWV and hypertension is further illustrated in Fig. 2a and b, respectively.

### Prognostic relevance of aortic PWV

Follow-up data was available in 401 patients (7 patients were lost to follow up, 1.7%). Median follow-up time was 13 (IQR 12–31) months. During follow-up period, MACCE was experienced by 44 (10.8%) patients, including 13 (3.2%) new congestive heart failures, 14 (3.4%) myocardial re-infarction, 8 (2.0%) strokes and 9 (2.2%) deaths. To determine the prognostic value of PWV in respect to established prognostic factors in STEMI, separate multivariable models for (A) patient characteristics, (B) CMR data and (C) biomarker data were conducted as described previously [22]. In multivariable Cox regression analysis, median PWV [6.6 m/s (IQR 5.6–8.3 m/s)] significantly and independently predicted the occurrence of MACCE after adjustment for patient characteristics [hazard ratio (HR) 2.45, 95% CI, 1.19–5.04, p = 0.014], CMR data (HR 2.77, 95% CI, 1.35–5.65, p = 0.005), and biomarker data (HR 2.62, 95% CI, 1.28–5.35, p = 0.008) (Table 4). Also, when applying the proposed cut-off value of 7.3 m/s, derived in an early analysis of the MARINA-STEMI cohort [4], PWV remained an independent predictor for MACCE (HR 2.71, 95% CI 1.44–5.11, p = 0.002) (Supplementary Table 5). The association of PWV and MACCE is further illustrated in Fig. 2c.

**Table 4:** Cox Regression Analysis for the Prediction of MACCE

	Univariable		Multivariable	
	HR (95% CI)	p-value	HR (95% CI)	p-value
**Model A: patient characteristics**				
Age, > 57 years	2.57 (1.34–4.91)	**0.004**		
Hypertension	3.53 (1.69–7.36)	**0.001**	2.85 (1.35–6.02)	**0.006**
Diabetes mellitus	2.68 (1.32–5.43)	**0.006**		
PWV, > 6.6 m/s	3.12 (1.54–6.33)	**0.002**	2.45 (1.19–5.04)	**0.014**
**Model B: CMR data**				
IS, > 15.2% LVMM	1.93 (1.02–3.62)	**0.043**		
LVEF baseline, < 52.4%	2.43 (1.30–4.54)	**0.006**	2.01 (1.10–3.92)	**0.024**
MVO	1.93 (1.02–3.65)	**0.044**		
PWV, > 6.6 m/s	3.12 (1.54–6.33)	**0.002**	2.77 (1.35–5.65)	**0.005**
**Model C: biomarker data**				
Peak hs-cT > 5035 ng/L	1.94 (1.04–3.63)	**0.037**		
Peak NT-proBNP > 1146 ng/L	2.96 (1.54–5.69)	**0.001**	2.57 (1.33–4.97)	**0.005**
Peak CK > 1945 U/L	1.61 (0.88–2.93)	0.122		
PWV, > 6.6 m/s	3.12 (1.54–6.33)	**0.002**	2.62 (1.28–5.35)	**0.008**

All p-values < 0.05 are highlighted in bold

*MACCE* major adverse cardiac and cerebrovascular events, *HR* Hazard ratio, *CI* confidence interval, *PW*V Pulse wave velocity, *IS* infarct size, *LVMM* left ventricular mass, *LVEF* left ventricular ejection fraction, *MVO* microvascular obstruction, *Hs-cT* high-sensitivity cardiac Troponin T, *NT-proBNP* N-terminal prohormone of brain natriuretic peptide, *CK* Creatine kinase

## Discussion

In a large contemporary cohort of STEMI patients, we found that aortic stiffness, as determined by PWV using phase contrast CMR imaging, was independently associated with age, hypertension, and multivessel disease. In contrast, other conventional cardiovascular risk factors including sex, diabetes, smoking status, dyslipidemia, and obesity did not show a significant association in adjusted analysis. Therefore, in patients with recent STEMI, aortic stiffness seems mainly dependent on age and increased blood pressure. In addition, we could demonstrate that STEMI patients with increased aortic stiffness have higher MACCE rates at medium term follow-up (13 months) and thus, in theory, might benefit from further therapeutic interventions that address key determinants of aortic stiffness.

Although there is solid evidence describing the determinants and prognostic implications of aortic stiffness in the general population [7, 23], data in patients post STEMI are scarce [3, 4]. Our study significantly expanded these previous data by demonstrating that, in STEMI patients, aortic stiffness is mainly dependent on age and increased blood pressure. Importantly, we could also show that increased aortic stiffness is associated with worse clinical outcomes independent of other clinical risk factors or CMR parameters. As such, this study provides further insights in the pathology of increased aortic stiffness in the largest STEMI cohort so far and adds important insights in prognostication and possible future management using PWV as a solid long-term biomarker for blood pressure control to identify STEMI patients at risk to develop MACCE.

### Aortic stiffness and aging

Chronological aging represents an unmodifiable risk factor and has a major influence on the cardiovascular system. One of the most evident effects is the development of atherosclerosis and stiffening of large arteries [24]. Accordingly, age is considered a major independent and unmodifiable determinant of increased aortic stiffness [7]. In our cohort, age was strongly correlated with aortic stiffness as determined by PWV. Multivariable analysis revealed that age was associated with PWV independently of other cardiovascular risk factors. The degree of stiffening of large arteries is strongest for people between 50 and 70 years of age [25]. Median age of our study cohort was 57 years, hence, might be an explanation for the very strong association of PWV with age observed in our cohort.

### Aortic stiffness and cardiovascular risk factors

There is a general lack of consistency between studies if arterial stiffening is also accelerated in the presence of modifiable cardiovascular risk factors such as diabetes, dyslipidemia, obesity and tobacco smoking [7]. After STEMI, these factors are prognostically important [26–30] and a correlation with aortic stiffness could be a possible pathophysiological explanation. However, in concordance to other studies [7, 31], we could not demonstrate a significant correlation between PWV and diabetes, dyslipidemia or obesity after multivariable adjustment. Therefore, aortic stiffening seems not a major mechanistic explanation for the relationship of these risk factors and outcome in STEMI patients.

### Aortic stiffness and hypertension

At the present state of knowledge it is unclear whether hypertension promotes arterial stiffening or vice versa [32]. Due to the fact that there are observations that speak both for and against the hypothesis that hypertension is rather cause than consequence, it is most likely that both hypertension, as well as, arterial stiffening mutually influence each other [32, 33]. In most studies hypertension is, however, a well-described determinant of arterial stiffening [7]. In the present study, we confirm these results also for patients after acute STEMI. In multivariable regression analysis, hypertension significantly and independently predicted aortic stiffness. Our findings are in line with previous studies across multiple subpopulations, which observed strongest relationship between age and hypertension, whereas other cardiovascular risk factors failed to reliably predict arterial stiffening [7]. Higher PWV in patients with hypertension and STEMI could therefore in part explain the increased risk for adverse outcome in STEMI patients with hypertension [34].

### Aortic stiffness and multi-vessel coronary artery disease

Another finding of our study is that in multivariable regression analysis, the number of affected vessels, detected at the time of PPCI, was significantly and independently associated with PWV. This observation underscores the validity of our data and is in line with previous findings, where a close relationship between PWV and the extent of coronary artery disease has been observed [35, 36].

### Aortic stiffness and prognosis

Earlier studies demonstrated the prognostic value of aortic stiffness in the general population [23]. In this study, we were able to prove these findings also for patients with STEMI, as PWV significantly and independently predicted MACCE after adjustment for patient characteristics, CMR data and biomarker data. Furthermore, we could corroborate the prognostic relevance of the previously suggested cut-off value of 7.3 m/s [4], in a more than two times larger cohort.

Considering our findings, the association between MACCE and PWV is mainly driven by the occurrence of myocardial re-infarction (3.4%) and new congestive heart failure (3.2%). This is in line with previous findings, demonstrating that increased arterial stiffening and their related changes in hemodynamics can cause vascular shear stress resulting in atherosclerotic plaque rupture and subsequent myocardial infarction [37, 38]. Additionally, our findings emphasize the pathophysiological impact of aortic stiffness on the myocardium resulting in new congestive heart failure. This may be mainly caused by myocardial fibrosis, left ventricular hypertrophy and hampered myocardial perfusion which are known to be of importance in the setting of adverse cardiac remodeling and LV dysfunction [1, 39]. Stroke was responsible for 2.2% of MACCE. These findings are in line with previous data indicating that arterial stiffening increases the risk for subclinical brain infarction and incident of stroke [40]. This may be caused by high aortic pulsatility and related changes in hemodynamics, leading to plaque ulceration, brain vascular remodeling and impaired oxygen delivery to the brain [1, 37].

Due to its prognostic implications [4, 23], knowledge about main factors influencing aortic stiffness might assist to further optimize measures of secondary prevention. Per definition, aging, as an unmodifiable risk factor does not allow any preventive interventional strategies. Hypertension, however, as the other main determinant of arterial stiffening offers a valuable therapeutic target for antihypertensive agents. Considering, that the progression of arterial stiffening is thought to be non-linear and peaks within 50–70 years of age [25, 31], in younger STEMI patients, hypertension represents the main modifiable determinant of arterial stiffening and provides an effective therapeutic target for several antihypertensive drugs which have shown to reduce stiffening of large arteries over time [10]. In respect of the tight relationship between hypertension and PWV demonstrated by our data and by the ongoing debate if hypertension is rather cause than consequence of arterial stiffening [32], it has to be assumed that hypertension and aortic stiffness positively influence each other. Due to the fact that at present we cannot target aortic stiffness as there is no therapeutic approach available, unfavorable factors that strongly influence aortic stiffness have to be eliminated. Hence, strict antihypertensive therapy (especially in high risk STEMI patients with increased PWV values), as emphasized by the SPRINT Trial [41], might offer the potential to reduce ongoing arterial stiffening, prolong lifespan [42], prevent major cardiovascular complications [41] and in a further consequence improve clinical outcome [4].

This study shows that aortic stiffness should not be considered as an innocent expression of vascular aging but as relevant marker of adverse cardiovascular outcome in patients with STEMI. Further work, preferable randomized studies, should focus on the exact role of aortic stiffness in risk stratification and therapeutic guidance in this population of high-risk patients.

### Limitations

The present study has limitations. Firstly, availability of cardiovascular risk factors before the cardiac event, such as detailed information on hypertension including the grade of hypertension or data on 24-h blood pressure behavior, as well as long-term blood-sugar levels were missing. Hence, discrimination between patients with moderate to high-risk and their impact on arterial stiffening could not be performed. Comparable with other studies, twelve percent of the patients in our analysis had diabetes [43]. However, this relatively small number of diabetic patients limits definitive conclusions regarding the association between PWV and diabetes in STEMI patients.

Secondly, the exclusion criteria of our study may have led to an underrepresentation of high-risk patients such as older patients with advanced comorbidities. Nevertheless, our analysis included a broad range of consecutive STEMI patients treated according to contemporary guidelines. The median age and other baseline characteristics are comparable with previous data on all-comer STEMI patients [44] and large multicenter CMR STEMI studies [45, 46]. Although our analysis is currently the largest CMR study on the prognostic role of PWV after STEMI, the relative small number of adverse events limited the number of variables that could be included in the multivariable models. Therefore, further validation is desirable.

## Conclusion

Our findings show that age and hypertension are the key determinants of aortic stiffness in patients with recent STEMI. In contrast, sex, diabetes, smoking status, dyslipidemia, and obesity did not show a significant association with aortic stiffness. Furthermore, we could demonstrate that patients with recent STEMI and increased aortic stiffness have higher rates of MACCE events. Considering that hypertension might be the major modifiable determinant of arterial stiffening, PWV could be viewed as a long-term biomarker for blood pressure control to identify STEMI patients at an increased risk for the development of MACCE. In this specific population, it should be of major interest to aggressively control blood pressure to potentially prolong lifespan [42] and improve clinical outcome [4]. Further research in this direction is warranted.

## Supplementary Information

Below is the link to the electronic supplementary material.

Supplementary file1 (DOCX 18 kb)

## Data Availability

The data that support the findings of this study are available from the corresponding author on reasonable request.

## Declarations

### Availability of data and material

The authors have full control of all primary data. The data underlying this article will be shared on reasonable request to the corresponding author.
