# Supplementary material for: Determinants and prognostic relevance of aortic stiffness in patients with recent ST-elevation myocardial infarction
Source: Int J Cardiovasc Imaging. 2021 Sep 2;38(1):237–47. doi: 10.1007/s10554-021-02383-0 (PMC8818631; doi:10.1007/s10554-021-02383-0)
Supplement: Supplementary file 1 — Supplementary file1 (DOCX 18 kb) [file 10554_2021_2383_MOESM1_ESM.docx]

# Supplements

Table 5. Logistic Regression Analysis for Prediction of PWV ≥7.3 m/s

|  | **Univariable** |  | **Multivariable** |  |
| --- | --- | --- | --- | --- |
|  | **OR (95% CI)** | ***p-value*** | **OR (95% CI)** | ***p-value*** |
|  |  |  |  |  |
| **Age, years** | 1.12 (1.09-1.15) | **<0.001** | 1.12 (1.09-1.15) | **<0.001** |
| **Female sex** | 0.47 (0.28-0.80) | **0.006** | … |  |
| **Hypertension** | 3.05 (2.01-4.63) | **<0.001** | 2.32 (1.42-3.79) | **0.001** |
| **Current smoker** | 0.54 (0.36-0.81) | **0.003** | … |  |
| **Hyperlipidemia** | 0.94 (0.63-1.41) | 0.771 |  |  |
| **Diabetes mellitus** | 1.62 (0.88-2.99) | 0.120 |  |  |
| **Peak NT-proBNP** | 1.00 (1.00-1.00) | **0.001** | … |  |
| **Number of diseased vessels** | 1.50 (1.12-1.99) | **0.006** | … |  |

*PWV=Pulse wave velocity; NT-proBNP=N-terminal prohormone of brain natriuretic peptide; OR=Odds ratio; CI=Confidence interval.*
